# Characterization of the Prognostic Values of the CXCR1-7 in Clear Cell Renal Cell Carcinoma (ccRCC) Microenvironment

**DOI:** 10.3389/fmolb.2020.601206

**Published:** 2020-11-25

**Authors:** Zhulin Wu, Yingzhao Zhang, Xiang Chen, Wanjun Tan, Li He, Lisheng Peng

**Affiliations:** ^1^The Fourth Clinical Medical College of Guangzhou University of Chinese Medicine, Shenzhen, China; ^2^Shenzhen Futian Center for Chronic Disease Control, Shenzhen, China; ^3^Department of Oncology and Haematology, Shenzhen Traditional Chinese Medicine Hospital, Shenzhen, China; ^4^Department of Science and Education, Shenzhen Traditional Chinese Medicine Hospital, Shenzhen, China

**Keywords:** CXCR, clear cell renal cell carcinoma (ccRCC), tumor microenvironment, bioinformatics analysis, prognosis

## Abstract

**Background:** As cancer immunotherapy has become a hot research topic, the values of CXC chemokine receptors (CXCRs) in tumor microenvironment have been increasingly realized. More and more evidence showed that the aberrant expression of CXCRs is closely related to the prognosis of various cancers. However, prognostic values and the exact roles of different CXCRs in clear cell renal cell carcinoma (ccRCC) have not yet been elucidated.

**Methods:** To further evaluate the potential of seven CXCRs as prognostic biomarkers for ccRCC, multiple online analysis tools, including ONCOMINE, UALCAN (TCGA dataset), Kaplan–Meier Plotter, MethSurv, cBioPortal, GEPIA, Metascape, and TIMER databases, were utilized in our research.

**Results:** The mRNA expression of CXCR4/6/7 was significantly increased in ccRCC patients, and all CXCRs are remarkably related to tumor stage or grade of ccRCC. Higher levels of CXCR3/4/5/6 expression were correlated with worse overall survival (OS) in patients with ccRCC, while higher expression of CXCR2 was associated with better OS. 23.14% mutation rate (118/510) of CXCR1-7 was observed in ccRCC patients, and the genetic alterations in CXCRs were related to worse OS and progression-free survival in ccRCC patients. Additionally, 53 CpGs of CXCR1-7 showed significant prognostic values. For functional enrichment, our results showed that CXCRs and their similar genes may be involved in cancer-associated pathways, immune process, and angiogenesis, etc. Besides, CXCRs were significantly correlated with multiple immune cells (e.g., CD8+ T cell, CD4+ cell, and dendritic cell).

**Conclusion:** This study explored the potential prognostic values and roles of the CXCRs in ccRCC microenvironment. Our results suggested that CXCR4 and CXCR6 could be the prognostic biomarkers for the patients with ccRCC.

## Introduction

Renal cell carcinoma (RCC) is the most common malignant tumor of kidney, and it is estimated that 350,000 people are diagnosed with RCC each year (Capitanio and Montorsi, 2016). Early RCC usually has a survival rate of 60–70% after treatment, but advanced RCC has a poor prognosis with 5-year survival of <10% (Dimitrieva et al., 2016). Additionally, clear cell renal cell carcinoma (ccRCC) is the most common type of renal cancer, and ccRCC accounts for ~75% of all cases of RCC (Lu et al., 2018). Despite continuous advances in cancer treatments, the mortality rate of ccRCC is still rising (He et al., 2018). Thus, new prognostic markers and therapeutic targets are urgently needed.

Recently, cancer immunotherapy has become a hotspot, and the values of CXC chemokine receptors (CXCRs), crucial components of the immune system, have been increasingly reported. CXCRs are a group of cell surface G-protein coupled receptors, and CXCR family members in tumor microenvironment serve as regulators of cancer progression by binding to unique ligands. To date, seven CXCR family members (CXCR1-7) have been identified. It has been reported that CXCRs and their ligands could affect tumor cell activation, proliferation, invasion, and migration (Zhu et al., 2012). Angiogenesis and the functions of tumor infiltrating immune cells are also related to CXCRs. Furthermore, previous studies have investigated the expression and prognostic values of some CXCR family members in different cancer tissues. For instance, prior research revealed that CXCR4 up-regulation represented an independent prognostic indicator for poor survival in RCC patients (Chen et al., 2014). A previous study showed that high expression levels of CXCR4 and CXCR7 predicted worse prognosis in patients with RCC (D'Alterio et al., 2010). Moreover, previous studies have identified the expression and prognostic values of CXCRs in gastric cancer (Yu and Zhang, 2019) and breast cancer (Guo et al., 2019).

However, prognostic values of CXCRs in ccRCC have not been thoroughly investigated, and the roles of different CXCRs in the initiation and development of ccRCC are still unclear. In the present study, we aimed to analyze the expression, methylation, and mutation of distinct CXCRs and their correlation with clinic-pathological features and prognosis in ccRCC patients. Moreover, we also investigated the predicted functions of CXCRs and their similar genes. Besides, our additional goal was to analyze the relationship between CXCRs and components of tumor immune cell infiltration.

## Methods

### Analysis of ONCOMINE Datasets

Firstly, CXCR1/2/3/4/5/6/7 mRNA levels were analyzed using public datasets available on ONCOMINE (http://www.oncomine.org) (Rhodes et al., 2007). In our study, the following values were utilized as thresholds: top 10% gene rank, *p*-value was set to 0.01, data type was set to mRNA, and fold change (FC) was defined as 1.5. Then, the differences in expression between cancer tissues and corresponding normal samples for CXCR1/2/3/4/5/6/7 were compared.

### Analysis of TCGA Dataset Using UALCAN

The ccRCC data in The Cancer Genome Atlas (TCGA) database were analyzed using the UALCAN platform (http://ualcan.path.uab.edu). UALCAN is an online open-access platform that contains TCGA raw data, including gene expression and clinic-pathological data (Chandrashekar et al., 2017). In the present study, UALCAN database was employed to analyze the mRNA expression of seven CXCRs in ccRCC tissues and their relationship with clinicopathological parameters (cancer stage and grade). Besides, samples lacking cancer stage or grade information were excluded from the corresponding analyses.

### Survival Analysis Using the Kaplan–Meier Plotter

The prognostic values of CXCR1-7 and combinatory mRNA expression of seven CXCRs in ccRCC were evaluated using an online database, Kaplan-Meier (KM) Plotter (http://www.kmplot.com) (Nagy et al., 2018). The KM Plotter comprises gene expression profiles and survival information of 21 cancer types, including survival data and mRNA expression of 530 ccRCC patients. The survival outcome was overall survival (OS), and the optimal cutoff value was determined by the algorithms in KM plotter.

### Analysis of Genetic Mutations in CXCRs Using cBioPortal

In the present study, genetic mutations in CXCRs and their correlation with OS and progression-free survival (PFS) of ccRCC patients were explored. The cBioPortal database (http://www.cbioportal.org) was utilized to analyze the genome profiles of seven CXCRs, which included mutations, putative copy-number alterations from Genomic Identification of Significant Targets in Cancer (GISTIC), and mRNA Expression z-Score (RNASeqV2RSEM) with a score threshold of ±1.8. The cBioPortal is an online tool for exploring and visualizing multidimensional cancer genome datasets (Gao et al., 2013). Also, KM plots were applied to assess the relationship between Genetic mutations in CXCRs and survival time of ccRCC patients using cBioPortal.

### DNA Methylation Information of CXCRs in MethSurv

MethSurv database (https://biit.cs.ut.ee/methsurv/) was applied to analyze the DNA methylation sites of CXCR family members in TCGA. The MethSurv is an open-access tool for multivariable survival analysis of DNA methylation data (Modhukur et al., 2018). Moreover, the prognostic values of CpG methylation in CXCR1-7 were evaluated, and the survival outcome was OS.

### Functional Enrichment Analyses of CXCRs and Their Similar Genes

Before performing functional enrichment analyses, the similar genes of each CXCR family member were obtained using the Gene Expression Profiling Interactive Analysis platform (GEPIA, http://gepia.cancer-pku.cn/). GEPIA database can provide analysis functions, including gene differential expression analysis, correlation analysis, and detection of similar genes, etc. (Tang et al., 2017). In addition, correlation analysis of CXCR1-7 was performed in the light of online instructions of GEPIA Correlation Analysis, and visualization of the results was achieved using a bioinformatics online platform from China (http://www.bioinformatics.com.cn/). Furthermore, Metascape (http://metascape.org) was used to perform Gene Ontology (GO) and Kyoto Encyclopedia of Genes and Genomes (KEGG) analyses of CXCR1-7 and their similar genes. Metascape is a comprehensive website for gene annotation and enrichment analysis, which is updated monthly to ensure that its content is up to date (Zhou et al., 2019). In this study, the thresholds of the Min Overlap, *P*-value, and Min Enrichment in Metascape were set to 3, 0.05, and 3, respectively. The term with the greatest statistical significance within a cluster was selected as the one representing the cluster. Besides, Protein-protein interaction (PPI) analysis was also conducted, and Molecular Complex Detection (MCODE) algorithm was utilized to identify network components with dense connections.

### Immune Infiltrates Correlation Analysis Using Timer

Associations between six immune infiltrates and CXCRs were analyzed by the Tumor Immune Estimation Resource tool (TIMER, https://cistrome.shinyapps.io/timer/). TIMER is an online tool for systematical analyses of immune infiltration of various cancers (Li et al., 2017). In this study, the purity-corrected partial Spearman's correlation (partial-cor) and *p*-value provided by TIMER were showed in scatterplots.

### Statistical Methods

Differences between two groups were compared by using the Student's *t*-test. Correlations were determined using Pearson or Spearman correlation tests, as appropriate. The survival curve was plotted by the KM method, with a hazard ratio (HR) with 95% confidence intervals and log rank *p*-value. A *p* < 0.05 was considered a statistically significant difference in all circumstances.

## Results

### Overexpression of Distinct CXCR Family Members in ccRCC Patients

**Figure 1:**
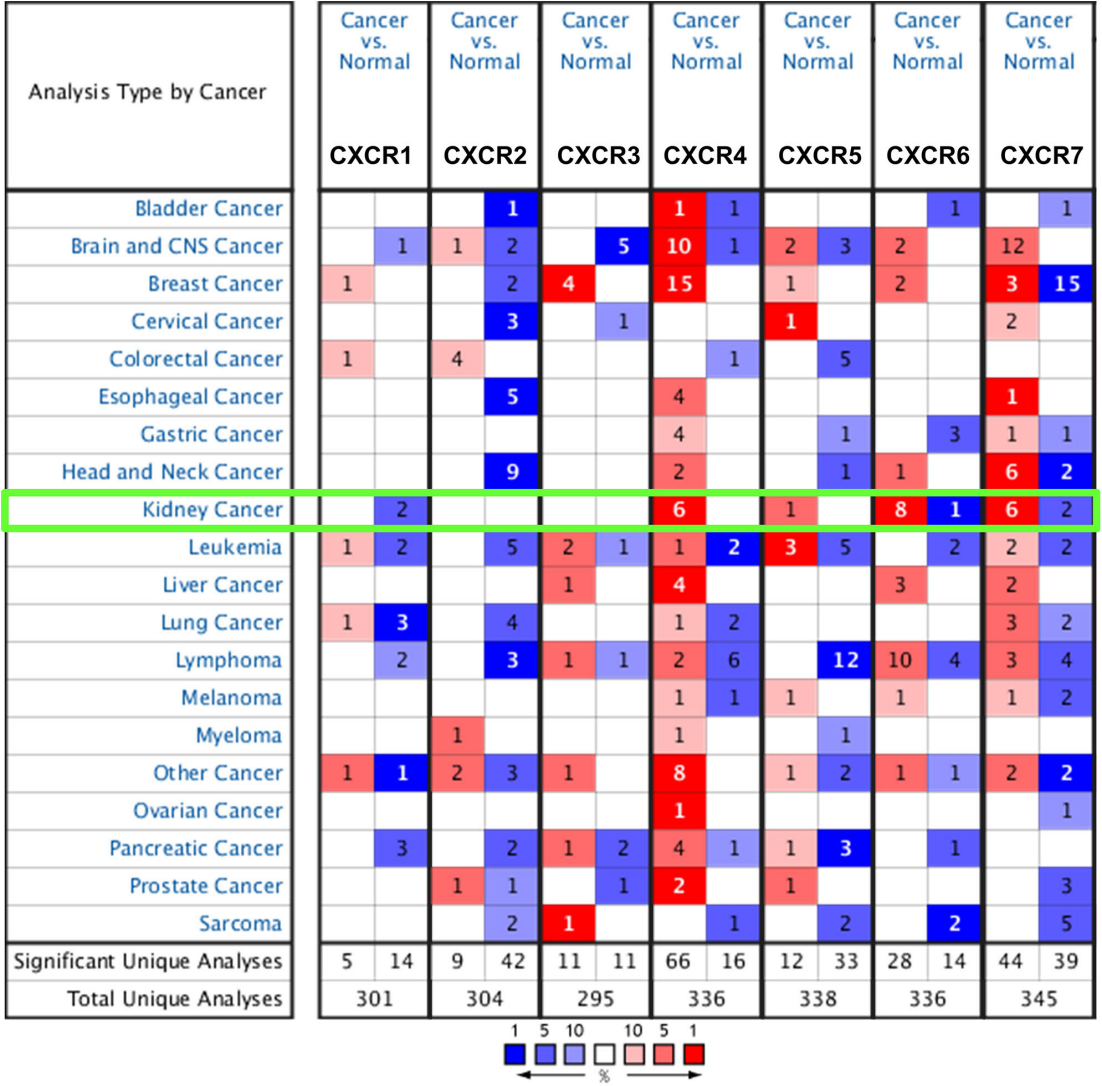
The mRNA expression of CXCRs (cancer vs. normal tissue of kidney) was assessed using the ONCOMINE database. Red represents significant overexpression and blue represents reduced expression. The number in each cell stands for the number of analyses that meet the threshold.

So far, seven CXCR family members have been identified in various cancers, including central nervous system (CNS) cancer, breast cancer, kidney cancer, etc (Figure 1). Also, the mRNA expression between ccRCC and normal tissue specimens was compared using the ONCOMINE (Table 1). Figure 1 showed that mRNA expression levels of CXCR4/5/6/7 were upregulated in all types of kidney cancers. As shown in Table 1, the mRNA levels of CXCR4 were prominently higher in ccRCC in six datasets (Lenburg et al., 2003; Jones et al., 2005; Gumz et al., 2007; Beroukhim et al., 2009; Yusenko et al., 2009). In Gumz dataset (Gumz et al., 2007), CXCR4 was overexpressed in ccRCC tissues compared with normal tissues with a FC of 6.895 (*p* = 9.24E-12; Gumz et al., 2007), while Jones found a 9.056-fold increase in CXCR4 mRNA expression in ccRCC specimens (*p* = 2.62E-20; Jones et al., 2005) and Lenburg observed 9.160-fold increase in CXCR4 mRNA expression in ccRCC tissues (*p* = 1.65E-4; Lenburg et al., 2003). In Beroukhim dataset (Beroukhim et al., 2009) and Yusenko dataset (Yusenko et al., 2009), the mRNA expression of CXCR4 in ccRCC was also higher than that in normal kidney tissues. Moreover, significantly higher mRNA expression of CXCR7 was found in ccRCC tissues in six datasets (Higgins et al., 2003; Lenburg et al., 2003; Jones et al., 2005; Gumz et al., 2007; Beroukhim et al., 2009). In Gumz dataset (Gumz et al., 2007), the FC of the expression of CXCR7 in ccRCC was 4.996 and a *p*-value of 1.85E-7. In Jones research (Jones et al., 2005), CXCR7 was overexpressed in ccRCC with a FC of 9.349 and a *p*-value of 9.07E-12. In Lenburg et al. (2003) and Higgins et al. (2003) studies, CXCR7 was prominently up-regulated in ccRCC with FC of 2.944 (*p* = 0.004) and 4.676 (*p* = 0.001), respectively. The transcriptional level of CXCR7 in ccRCC was remarkably different from that in the normal kidney tissues in Beroukhim's research (Beroukhim et al., 2009). In addition, Jones observed a 2.004-fold increase in the expression of CXCR6 in ccRCC specimens (*p* = 6.44E-16; Jones et al., 2005) and Yusenko found a 5.264-fold increase in the expression of CXCR6 in ccRCC samples (*p* = 3.66E-5; Yusenko et al., 2009). Besides, no significant difference was observed regarding CXCR1/2/3/5 mRNA expression in ccRCC.

**Table 1:** Transcriptional levels of CXCRs family members between normal kidney tissues and ccRCC (ONCOMINE).

**No**	**Gene name**	**Fold change**	***p*-value**	***t*-test**	**References**
1	CXCR4	6.895	9.24E-12	14.920	Gumz Renal (Gumz et al., 2007)
2	CXCR4	9.056	2.62E-20	16.516	Jones Renal (Jones et al., 2005)
3	CXCR4	9.160	1.65E-4	4.855	Lenburg Renal (Lenburg et al., 2003)
4	CXCR4	8.603	1.03E-6	7.613	Beroukhim Renal (Beroukhim et al., 2009)
5	CXCR4	10.066	1.03E-6	8.631	Beroukhim Renal 2 (Beroukhim et al., 2009)
6	CXCR4	3.801	7.28E-5	6.456	Yusenko Renal (Yusenko et al., 2009)
7	CXCR6	2.004	6.44E-16	12.195	Jones Renal (Jones et al., 2005)
8	CXCR6	5.264	3.66E-5	7.588	Yusenko Renal (Yusenko et al., 2009)
9	CXCR7	4.996	1.85E-7	7.776	Gumz Renal (Gumz et al., 2007)
10	CXCR7	9.349	9.07E-12	10.000	Jones Renal (Jones et al., 2005)
11	CXCR7	2.944	0.004	3.209	Lenburg Renal (Lenburg et al., 2003)
12	CXCR7	9.349	9.07E-12	10.000	Beroukhim Renal (Beroukhim et al., 2009)
13	CXCR7	6.654	1.64E-10	10.076	Beroukhim Renal 2 (Beroukhim et al., 2009)
14	CXCR7	4.676	0.001	7.821	Higgins Renal (Higgins et al., 2003)

### TCGA Dataset Analysis by UALCAN

**Figure 2:**
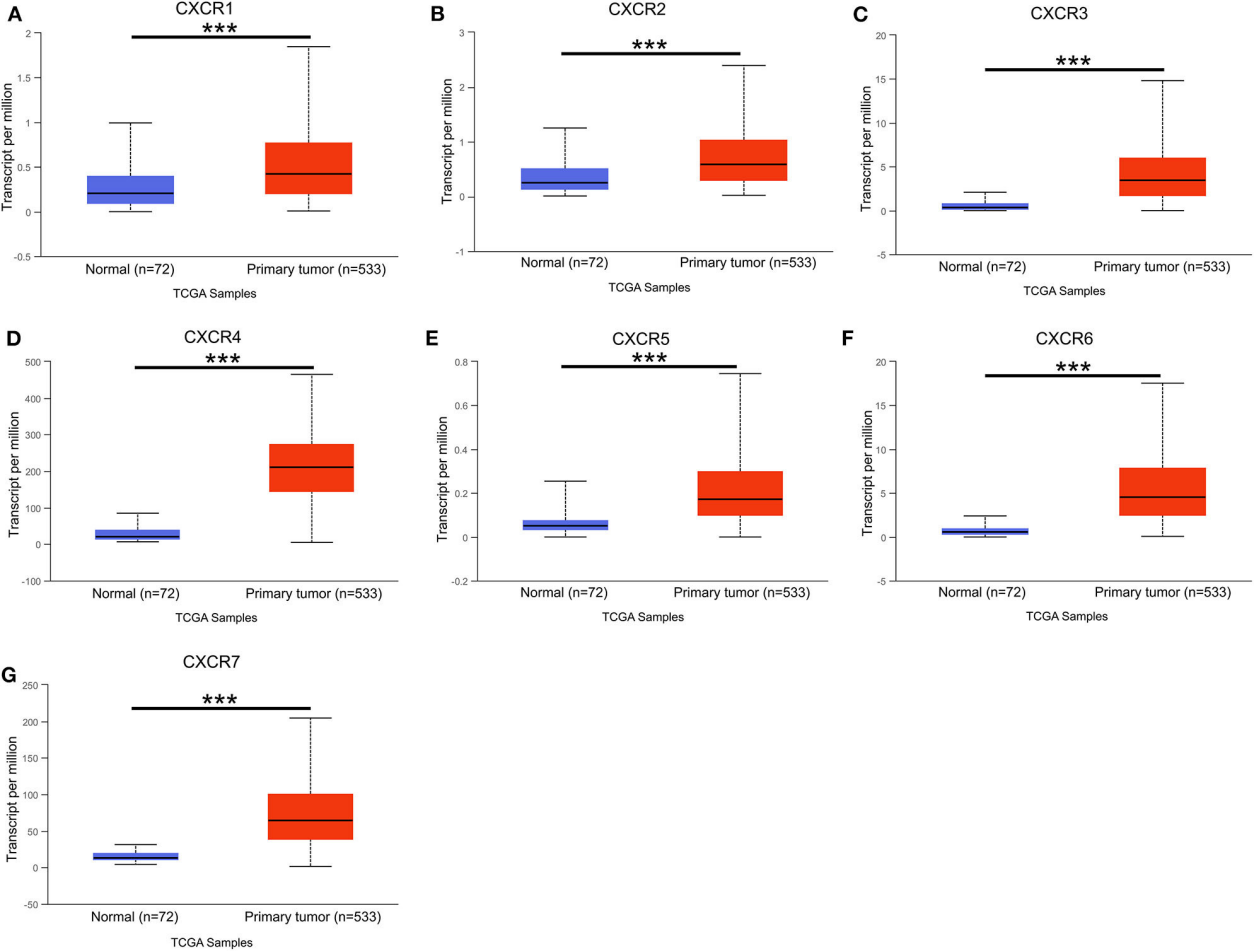
The mRNA expression of CXCR1-CXCR7 in normal kidney tissues (Normal) and ccRCC (Primary tumor) from the TCGA cohort (UALCAN). **(A–G)** seven CXCR family members mRNA expression levels were increased in the ccRCC compared with normal tissues. ***p < 0.001.

**Figure 3:**
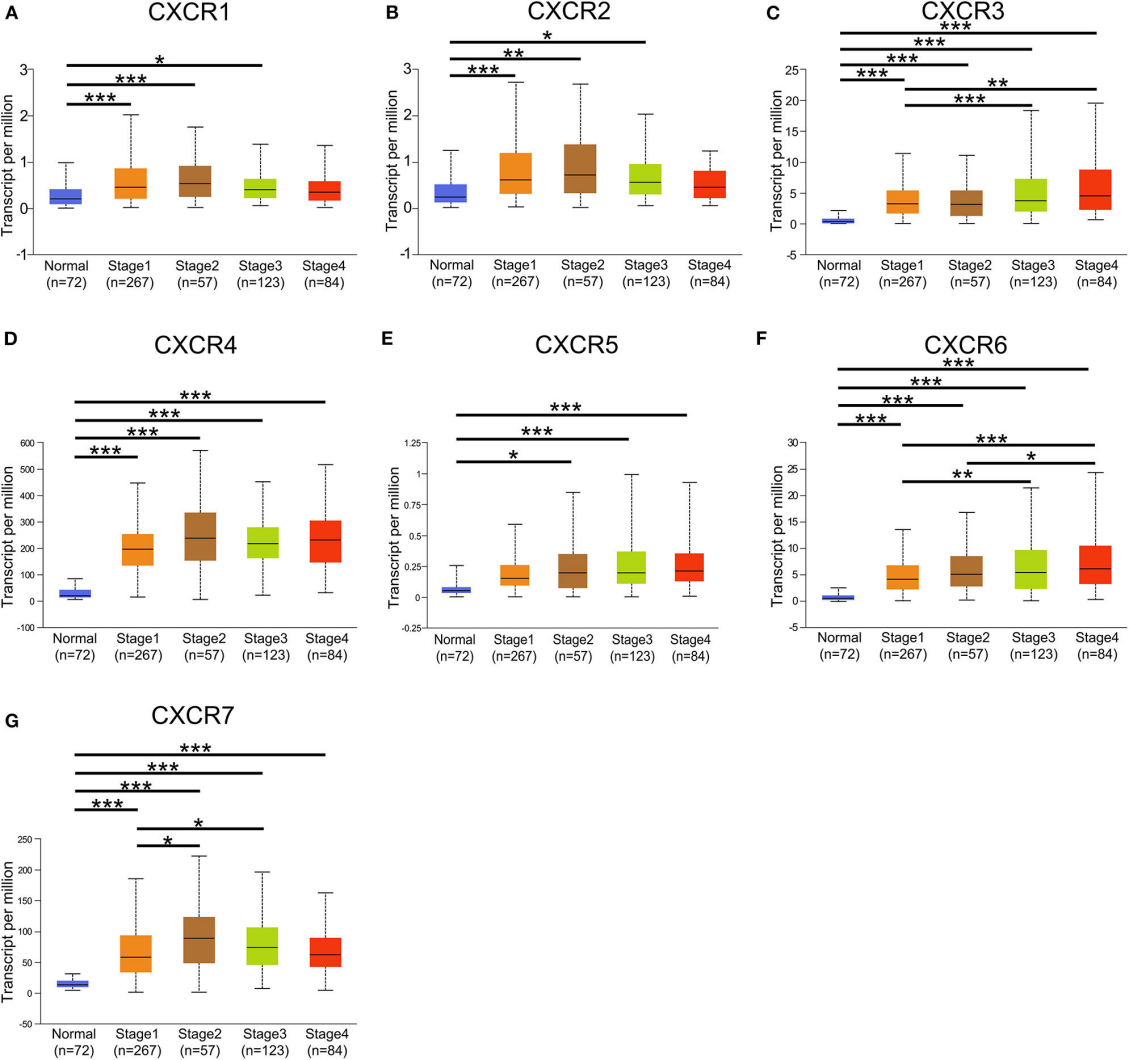
Correlation between mRNA expression of distinct CXCR1-CXCR7 and tumor stages in ccRCC analyzed using UALCAN. **(A–G)** mRNA expression of CXCR family members is significantly associated with the clinical stages of patients with ccRCC. The highest mRNA expression of CXCR1/2/4/7 was detected in stage 2 **(A,B,D,G)**, and the highest mRNA expression of CXCR3/5/6 was detected in stage 4 **(C,E,F)**. *p < 0.05, **p < 0.01, ***p < 0.001.

**Figure 4:**
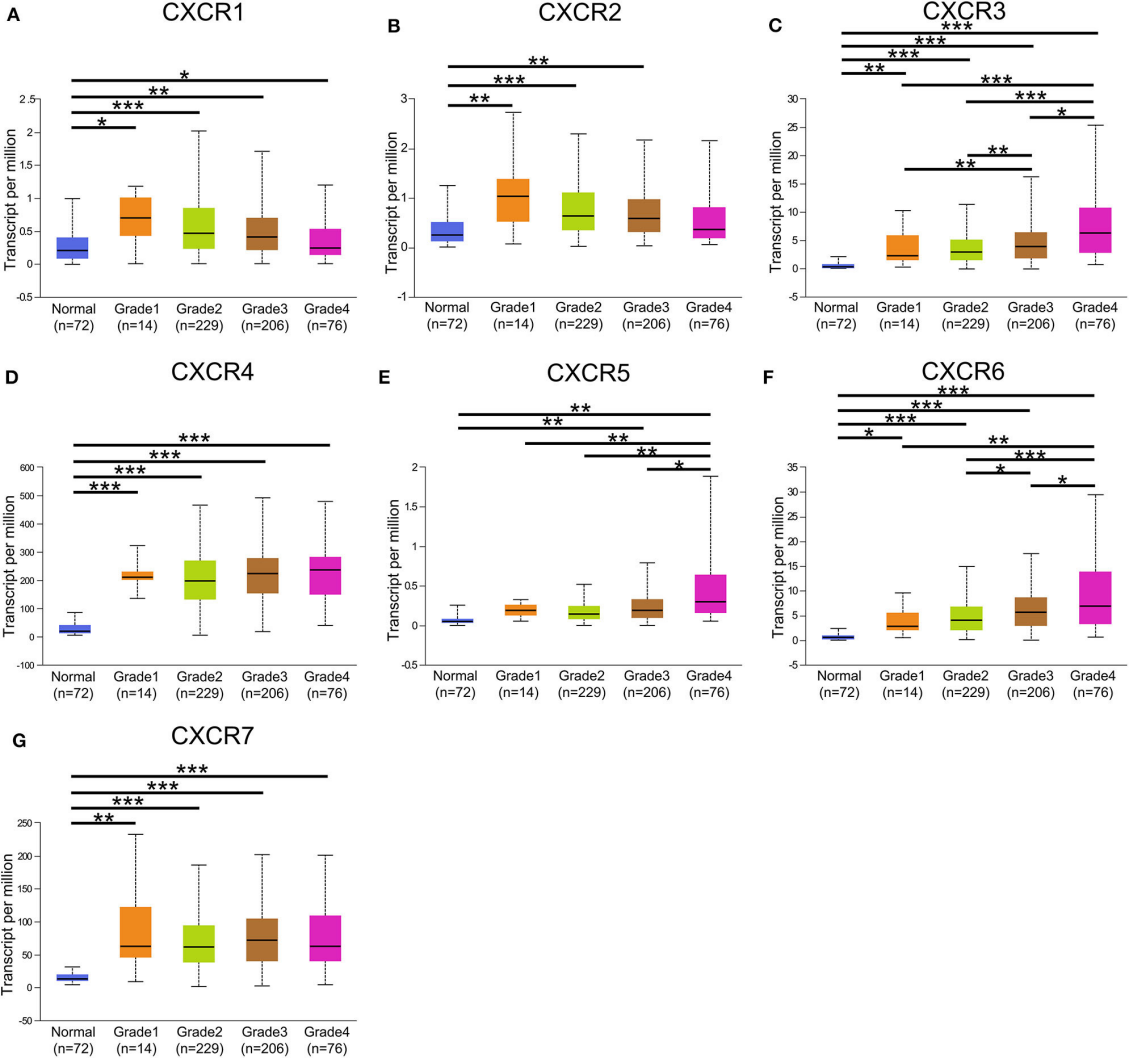
CXCR family genes expression in patients with different grades. **(A–G)** A significant correlation was found between the expression of CXCR family genes and pathological grade. The mRNA expression of CXCR7 was the highest in grade 3 **(G)**, while the highest mRNA expression of CXCR3/4/5/6 was found in grade 4 **(C–F)**. The highest mRNA expression of CXCR1/2 was detected in grade 1, and the mRNA expression of CXCR1/2 tended to decrease as grade increased **(A,B)**. *p < 0.05, **p < 0.01, ***p < 0.001.

In the present study, TCGA data of ccRCC were utilized to validate the expression patterns of the CXCR members. The results of UALCAN revealed that the mRNA expression of all CXCR family members was remarkably higher in ccRCC samples than in normal kidney tissues (Figure 2). By using the UALCAN database, association of CXCRs expression with stage and grade in ccRCC was also analyzed. Before conducting the analyses, two samples lacking stage information and eight samples without grade information were not included in the corresponding analysis. As illustrated in Figures 3A–G, expression of seven CXCR members was closely associated with cancer stages of ccRCC patients, and patients with late stages (stage 3–4) tended to have higher mRNA expression of CXCR3/5/6 (Figures 3C,E,F). The highest expression levels of CXCR1/2/4/7 were observed in stage 2 (Figures 3A,B,D,G), while the highest expression levels of CXCR3/5/6 were found in stage 4 (Figures 3C,E,F). However, the sample size of stage 2 and stage 4 was still relatively small, which was a factor affecting the correlation. Similarly, as displayed in Figures 4A–G, box plots showed that CXCRs mRNA expression was prominently associated with pathological grades. As pathological grade increased, the expression of CXCR3, CXCR5, and CXCR6 tended to be higher (Figures 4C,E,F). Conversely, the mRNA expression of CXCR1 and CXCR2 was found to be negatively correlated with the pathological grade for ccRCC (Figures 4A,B). Collectively, the results of UALCAN indicated that mRNA expression of CXCR1-7 in ccRCC patients was significantly correlated with clinicopathological parameters.

### Prognostic Values of CXCRs Members in ccRCC

**Figure 5:**
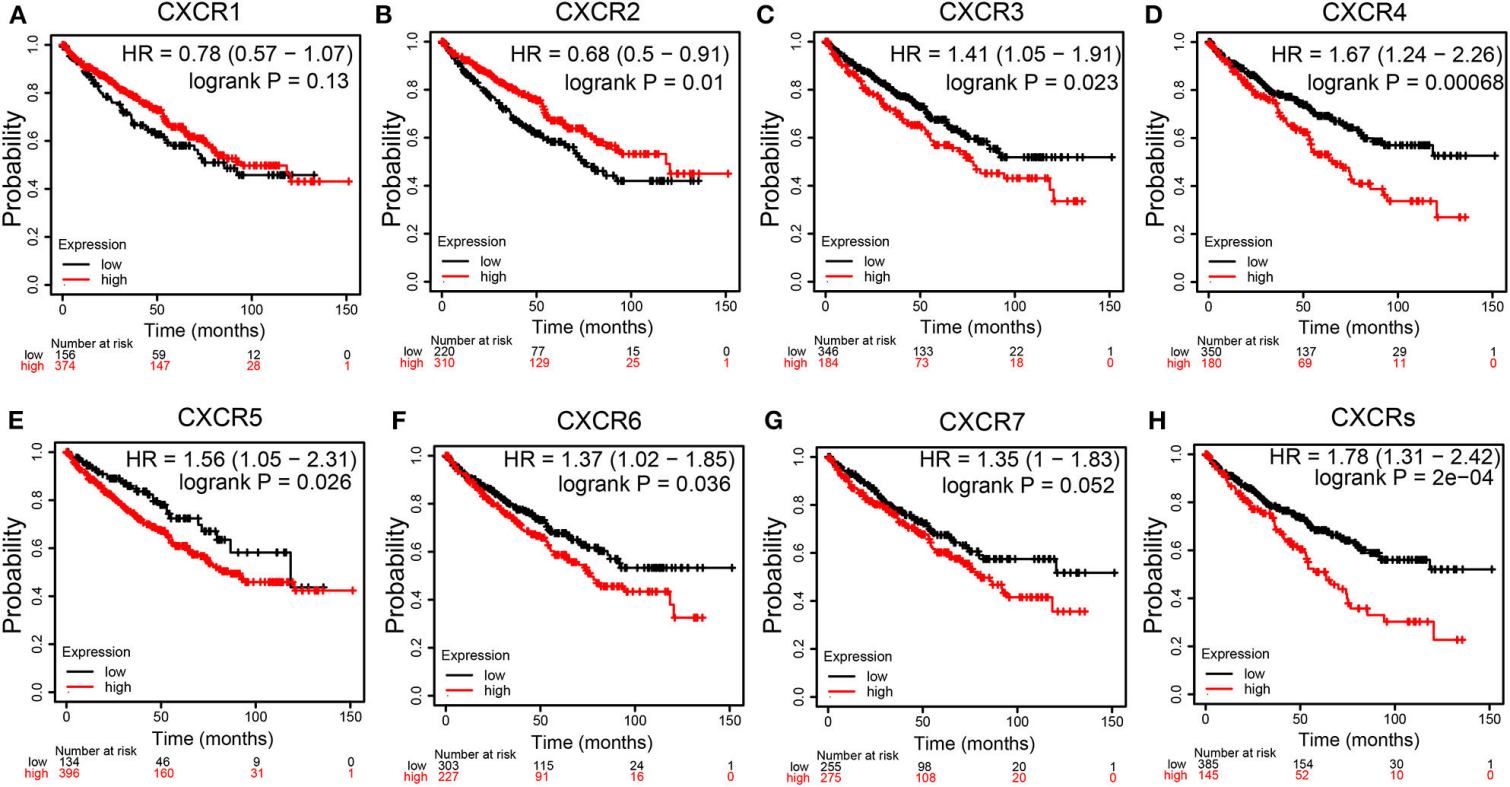
Survival curves showing the overall survival (OS) times of patients with ccRCC (Kaplan–Meier Plotter). **(A-G)** Kaplan–Meier (KM) survival curves of CXCR family members. Among the seven CXCR family members, high mRNA expression of CXCR3 **(C)**, CXCR4 **(D)**, CXCR5 **(E)**, CXCR6 **(F)**, and CXCRs **(H)** were associated with worse OS in ccRCC patients. High CXCR2 **(B)** were correlated with longer OS time. CXCR1 **(A)** and CXCR7 **(G)** showed no significant difference.

We assessed the prognostic values of CXCRs mRNA expression in patients with ccRCC using KM plotter (Figures 5A–G). As shown in Figures 5B–F, high mRNA expression levels of CXCR3 (*p* = 0.023), CXCR4 (*p* = 0.00068), CXCR5 (*p* = 0.026), and CXCR6 (*p* = 0.036) were significantly related to worse OS of patients with ccRCC, while high mRNA expression of CXCR2 (*p* = 0.01) were obviously associated with better OS time. The results also demonstrated that there were no associations between the mRNA expression of CXCR1 and survival (Figure 5A). Although not statistically significant, the KM curves showed that high CXCR7 expression levels may correlate with worse OS (Figure 5G). Furthermore, the correlation between the mean expression of all seven CXCRs and OS of ccRCC patients was analyzed (Figure 5H). The KM survival curve revealed that high combinatory mRNA expression of seven CXCRs was significantly related to shorter OS in patients with ccRCC (*p* = 2E-04).

### CXCRs Genetic Alterations in Patients With ccRCC

**Figure 6:**
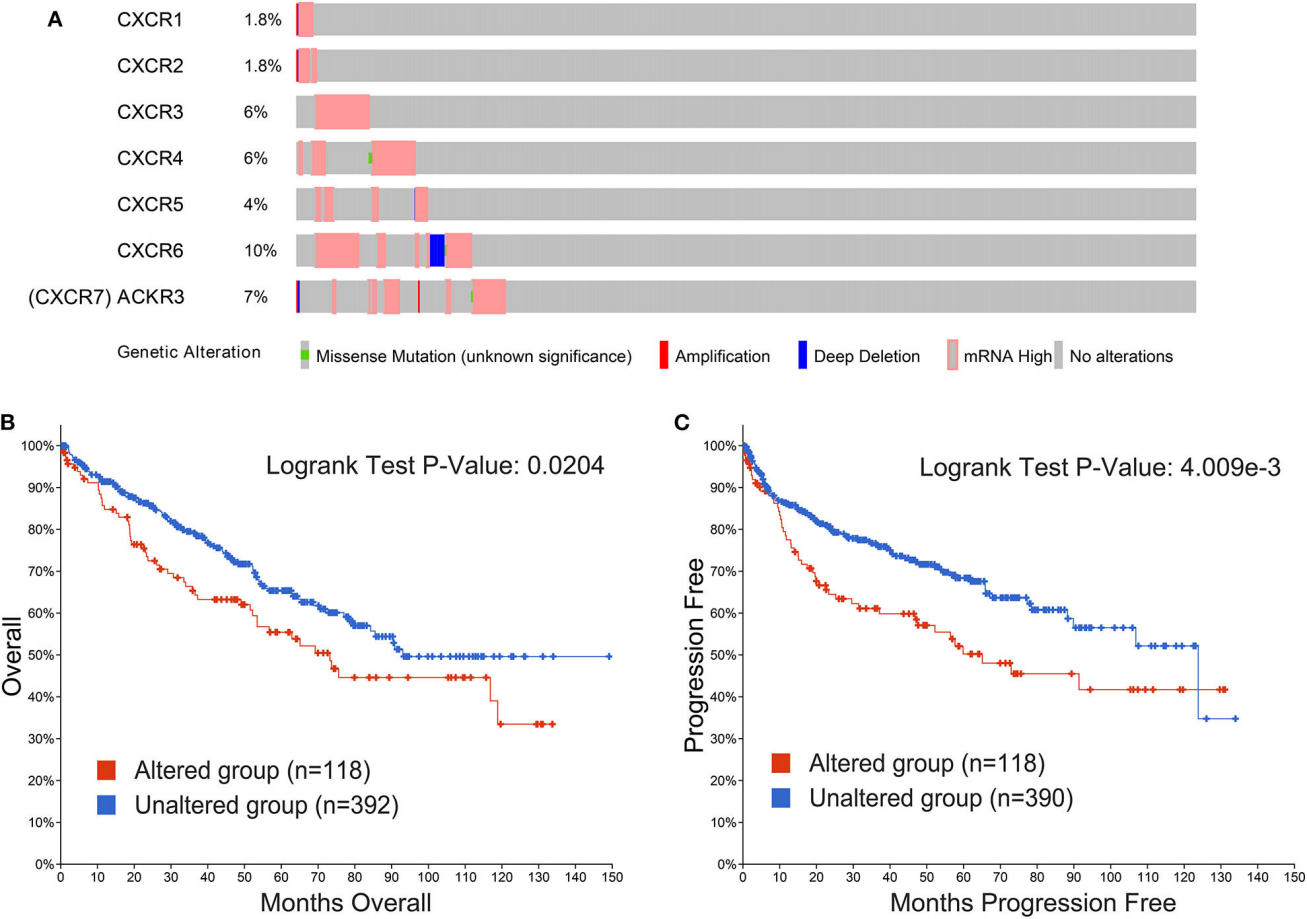
Alteration frequency of CXCRs and their correlation with overall survival (OS) and progression-free survival (PFS) of ccRCC patients. **(A)** The CXCRs mutation rate was 23.14% (118/510) in ccRCC patients. The top four highest mutation rates for CXCR family members were present in CXCR6 (10%), CXCR7 (CXCR7 is also called ACKR3, 7%), CXCR3 (6%), CXCR4 (6%), respectively. **(B)** Genetic alterations in CXCRs were significantly associated with OS of ccRCC patients (*p* < 0.05). **(C)** Genetic alterations in CXCRs were significantly related to PFS of ccRCC patients (*P* < 0.01).

Genetic alterations in CXCRs and their correlations with OS and PFS of ccRCC patients were explored by cBioPortal database. In our research, 510 ccRCC patients with CXCRs gene mutation information from the TCGA dataset were analyzed. As shown in Figure 6A, the percentages of genetic alterations in CXCRs of ccRCC ranged from 1.8 to 10% for single genes (CXCR1, 1.8%; CXCR2, 1.8%; CXCR3, 6%; CXCR4, 6%; CXCR5, 4%; CXCR6, 10%; CXCR7, 7%). The results of KM curves and log-rank test suggested that genetic alterations in CXCRs were correlated with worse OS (*p* = 0.0204) and PFS (*p* = 4.009e-3) of patients with ccRCC (Figures 6B,C). In short, genetic alterations in CXCRs could significantly influence the prognosis of ccRCC patients.

### Relationships Between CXCRs DNA Methylation and Prognosis of ccRCC

DNA methylation levels of CXCR family members with the prognostic value of each single CpG were investigated by the MethSurv tool. The results of MethSurv suggested that cg07016356 of CXCR1, cg14652717 of CXCR2, cg14296598 of CXCR3, cg07784959 of CXCR4, cg20208523 of CXCR5, cg05705212 of CXCR6, and cg05693814 of CXCR7 had the highest DNA methylation (Supplementary Figures 1, 2). Moreover, 7 CpGs of CXCR1, 4 CpGs of CXCR2, 1 CpG of CXCR3, 15 CpGs of CXCR4, 13 CpGs of CXCR5, 3 CpGs of CXCR6, and 10 CpGs of CXCR7 were related to the prognosis of ccRCC patients (*p* < 0.05, Table 2).

**Table 2:** The prognostic values of CpGs in CXCRs (*p* < 0.05).

**Gene-CpG**	**HR**	***p*-value**
CXCR1–5′UTR–Open_Sea–cg00832199	2.075	5e-04
CXCR1–5′UTR–Open_Sea–cg15768138	0.552	0.012
CXCR1–5′UTR–Open_Sea–cg15908708	0.606	0.011
CXCR1–Body–Open_Sea–cg09294937	1.623	0.013
CXCR1–3′UTR–Open_Sea–cg09905973	2.219	0.00087
CXCR1–TSS200–Open_Sea–cg18956547	0.585	0.0063
CXCR1–1stExon;5′UTR–Open_Sea–cg20025658	0.585	0.0077
CXCR2–5′UTR;1stExon–Open_Sea–cg06547715	1.861	0.012
CXCR2–TSS200;5′UTR–Open_Sea–cg13739417	0.606	0.025
CXCR2–5′UTR–Open_Sea–cg14652717	1.563	0.025
CXCR2–Body–Open_Sea–cg15657330	1.651	0.046
CXCR3–TSS200;Body–Open_Sea–cg17678039	0.541	0.0026
CXCR4–TSS200–N_Shore–cg12311057	0.465	0.00064
CXCR4–TSS200–N_Shore–cg20823742	0.624	0.041
CXCR4–Body;5′UTR;1stExon–N_Shore–cg02367708	3.019	6.6e-05
CXCR4–TSS1500–N_Shore–cg04513185	1.887	0.0013
CXCR4–TSS1500–N_Shore–cg06332859	2.799	0.00014
CXCR4–TSS1500–N_Shore–cg21859434	2.097	0.0032
CXCR4–3′UTR;1stExon–N_Shore–cg12595667	0.417	0.00073
CXCR4–Body;TSS1500–Island–cg02902079	0.578	0.021
CXCR4–Body;TSS1500–Island–cg10718991	2.247	0.0011
CXCR4–Body;TSS1500–Island–cg19238531	0.619	0.02
CXCR4–TSS1500–Island–cg06679534	1.589	0.048
CXCR4–TSS1500–Island–cg17398233	2.118	0.00014
CXCR4–TSS1500–Island–cg20366284	0.599	0.021
CXCR4–1stExon;5′UTR–Island–cg25982140	0.505	0.00056
CXCR4–Body–Island–cg22376465	1.868	0.0022
CXCR5–Body;TSS200–Open_Sea–cg03523129	2.539	4e-06
CXCR5–Body;TSS200–Open_Sea–cg19791714	2.074	0.0029
CXCR5–TSS1500–Open_Sea–cg01257799	0.67	0.042
CXCR5–Body;TSS1500–Open_Sea–cg03386765	1.992	0.0075
CXCR5–Body;TSS1500–Open_Sea–cg04537602	1.66	0.01
CXCR5–Body;TSS1500–Open_Sea–cg13298528	1.727	0.0061
CXCR5–TSS200–Open_Sea–cg04625873	3.272	3.3e-05
CXCR5–TSS200–Open_Sea–cg16235962	2.621	0.00018
CXCR5–TSS200–Open_Sea–cg17382048	3.247	2e-05
CXCR5–5′UTR;1stExon–Open_Sea–cg26164712	2.761	2.3e-05
CXCR5–1stExon; 3′UTR–Open_Sea–cg18728264	3.926	2.6e-06
CXCR5–1stExon;Body–Open_Sea–cg20208523	2.629	1.2e-05
CXCR5–1stExon;Body–Open_Sea–cg27049096	1.644	0.026
CXCR6–TSS1500;Body–Open_Sea–cg05705212	0.61	0.017
CXCR6–TSS200;Body–Open_Sea–cg25226014	0.599	0.012
CXCR6–3′UTR;Body–Open_Sea–cg26466027	0.525	0.001
CXCR7–5′UTR–S_Shore–cg00594866	0.536	0.0092
CXCR7–5′UTR–S_Shore–cg26960322	2.783	6.7e-05
CXCR7–TSS1500–S_Shore–cg27367871	3.071	3.9e-08
CXCR7–Body–Open_Sea–cg05693814	0.585	0.01
CXCR7–5′UTR–Open_Sea–cg15066967	3.55	3.7e-06
CXCR7–5′UTR–Open_Sea–cg17793354	2.51	5.2e-06
CXCR7–3′UTR–Open_Sea–cg27529004	0.416	8.8e-06
CXCR7–TSS1500–Island–cg07007118	0.603	0.012
CXCR7–TSS1500–Island–cg12088387	0.608	0.012
CXCR7–TSS1500–Island–cg12463851	0.57	0.014

### Functional Enrichment Analyses of CXCRs in ccRCC

**Figure 7:**
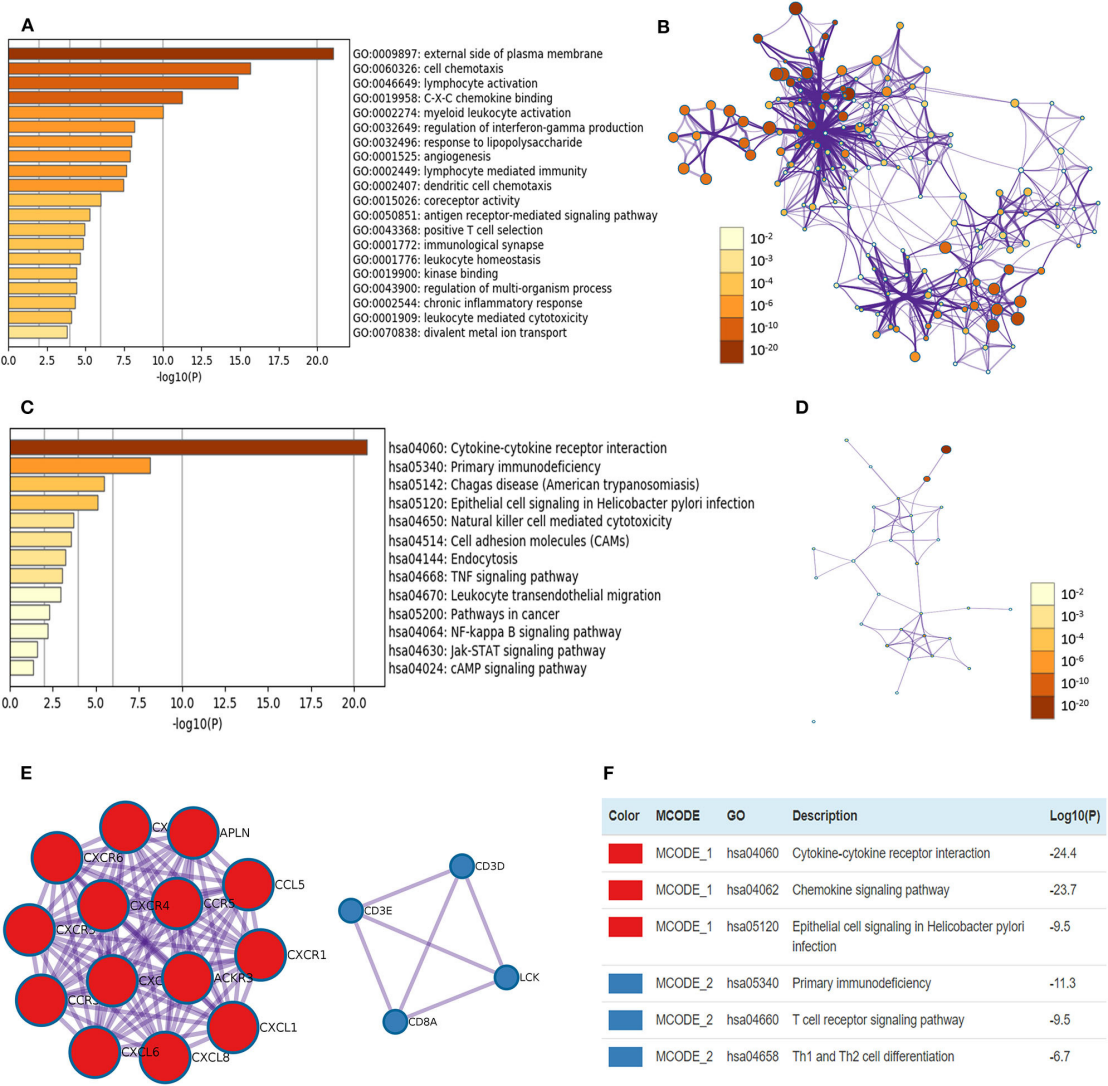
The enrichment analyses of CXCR1/2/3/4/5/6/7 and their similar genes in ccRCC. **(A)** GO enrichment analysis predicted three main functions, including biological process, cellular components, and molecular functions (top 20, *p* < 0.05), and each GO term is colored based on the value of -log10 (*p*-value). **(B)** The network of Enriched GO terms. Nodes represent GO terms, and node size indicates the number of genes involved. Nodes that share the same cluster are usually close to each other, and the thicker the edge, the higher the similarity. **(C)** KEGG pathways colored by *p*-values. **(D)** The network of KEGG pathways colored by *p*-value. **(E)** The two most significant MCODE components form the PPI network of CXCR family members and their similar genes. **(F)** Independent functional enrichment analysis of MCODE components.

Pearson correlation analysis of CXCRs in ccRCC was performed using GEPIA, and the data showed a statistically significant positive association among CXCR1 and CXCR2; CXCR3 with CXCR5 and CXCR6; CXCR4 with CXCR6 and CXCR7 (*R* > 0.2, Supplementary Figure 3). Additionally, the top 20 similar genes of each CXCRs family member were obtained (Supplementary Table 1). After removing duplicates, there were 117 genes, including seven CXCR family members and 110 similar genes. After that, the biological functions of seven CXCRs and their similar genes were explored by GO annotation and KEGG pathway analyses in Metascape. In this study, GO enrichment analysis included biological processes (BPs), cell components (CCs), and molecular function (MFs) terms (Figures 7A,B). As displayed in Figures 7A,B, CCs, including GO: 0009897 (external side of plasma membrane) and GO: 0001772 (immunological synapse) were significantly associated with CXCR family members and their similar genes. In addition, BPs such as GO: 0060326 (cell chemotaxis), GO:0046649 (lymphocyte activation), GO: 0002274 (myeloid leukocyte activation), GO: 0032649 (regulation of interferon-gamma production), and GO: 0032496 (response to lipopolysaccharide) were prominently related to CXCRs and their similar genes. Furthermore, CXCRs and their similar genes also remarkably affected MFs, such as GO: 0019958 (C-X-C chemokine binding), GO: 0015026 (coreceptor activity), and GO: 0019900 (kinase binding).

The top 13 KEGG pathways with *p* < 0.05 for the CXCRs and their similar genes are demonstrated in Figures 7C,D. Pathways such as has: 04060 (cytokine-cytokine receptor interaction), has: 05340 (primary immunodeficiency), has: 04650 (natural killer cell-mediated cytotoxicity), and has: 05200 (pathways in cancer) were correlated with the functions of CXCRs and their similar genes in ccRCC. Besides, the PPI network and the MCODE components of CXCRs and their similar genes are displayed in Figures 7E,F. The two most significant MCODE components were found from PPI analysis, and the results demonstrated that biological function was mostly associated with cytokine-cytokine receptor interaction (MCODE 1), chemokine signaling pathway (MCODE 1), T cell receptor signaling pathway (MCODE 2), and Th1 and Th2 cell differentiation (MCODE 2), etc.

### Tumor-Infiltrating Immune Cells Associated With CXCRs

**Figure 8:**
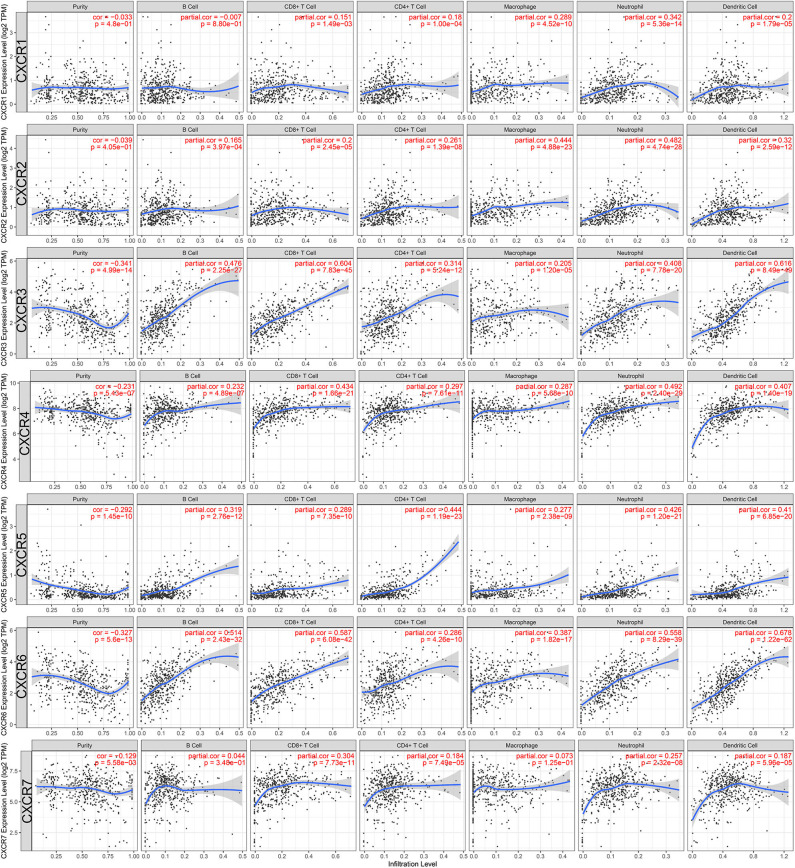
Associations between CXCR family members and tumor-infiltrating immune cells in ccRCC. The left-most panels show tumor purity. Also, the associations of tumor-infiltrating immune cells and CXCR1/2/3/4/5/6/7 in ccRCC were shown.

Last but not least, we also studied the potential immunological correlation between CXCRs and tumor-infiltrating immune cells. The expression levels of CXCR3/4/5/6/7 were negatively correlated with tumor purity (*p* < 0.05), suggesting that CXCR3/4/5/6/7 was highly expressed in the ccRCC microenvironment. Additionally, as shown in Figure 8, the association between B cell and CXCR6 (partial-cor = 0.514, *p* = 2.43E-32), CD8+ T cell and CXCR3 (partial-cor = 0.604, *p* = 7.83E-45), CD4+ T cell and CXCR5 (partial-cor = 0.444, *p* = 1.19E-23), macrophage and CXCR2 (partial-cor = 0.444, *p* = 4.88E-23), neutrophil and CXCR6 (partial-cor = 0.558, *p* = 8.29E-39), and dendritic cell and CXCR6 (partial-cor = 0.678, *p* = 1.22E-62) were comparably high.

## Discussion

At present, tumor microenvironment has become a research hotspot, in which the roles of chemokines and their receptors have received much attention. CXCRs in the cancer microenvironment serve as important regulators of cancer progression through binding to corresponding ligands. In our research, the expression levels of CXCR members in distinct cancers were analyzed, and we found that CXCR4, CXCR6, and CXCR7 were highly expressed in ccRCC samples from ONCOMINE. Additionally, CXCR1-7 were all highly expressed in ccRCC tissues of the TCGA dataset compared with normal tissues. Previous studies have shown that CXCRs are expressed at high levels in a variety of tumor tissues (Wang et al., 2018; Zheng et al., 2018; Jiang et al., 2019; Mir et al., 2019), but this study has, for the first time, summarized the expression of CXCR1-7 in ccRCC. Moreover, the expression of CXCRs mRNA in ccRCC was significantly correlated with cancer stage and pathological grade. In TCGA dataset, the expression of CXCR3/5/6 in ccRCC was positively correlated with the grade of malignancy or tumor stage, and the expression levels of CXCR1/2 were negatively associated with pathological grade. However, the highest expression of CXCR1/2/4/7 were observed in stage 2, and this may be relevant to the mechanism of lymph node and distant metastasis and the small sample size of some stage (stage 2 and 4).

In the present study, high expression of CXCR3/4/5/6 and high combinatory expression of seven CXCRs were related to poor survival outcomes in patients with ccRCC, while higher expression of CXCR2 was correlated with favorable survival in ccRCC patients. Furthermore, 23.14% mutation rate (118/510) of CXCR1-7 was observed in ccRCC patients and the genetic alterations in CXCR family members were related to worse OS and PFS in patients with ccRCC. Besides, although mRNA expression of CXCR1 and CXCR7 was not significantly correlated with OS in ccRCC, 53 CpGs of CXCR1-7 showed significant prognostic values. These results indicated that CXCR family members could be prognostic biomarkers for ccRCC patients. For functional enrichment, our results demonstrated that CXCRs and their similar genes may be involved in the immune process, angiogenesis, and tumor initiation and progression via a variety of signaling pathways (e.g., TNF pathway, pathways in cancer) and biological processes (e.g., angiogenesis). Also, we studied the association between tumor immune infiltrating cells and CXCRs. We found that all CXCR family members were significantly correlated with CD8+ T cell, CD4+ T cell, neutrophil, and dendritic cell, which were related to tumor progression, metastasis, or prognosis (Renner et al., 2017).

The results of TCGA analysis showed that CXCR1 and CXCR2 were highly expressed in ccRCC, and patients with advanced stages or grades tended to express lower mRNA expression of CXCR1/2. Additionally, a rather strong association was found between the expression of CXCR1 and CXCR2. It was reported that CXCR1 and CXCR2 have about 76% sequence homology and can bind to C-X-C Motif Chemokine Ligand (CXCL8) with similar affinities (Guo et al., 2019). It has been reported that overexpression of CXCR1/2 could promote metastasis of breast carcinoma (Kaunisto et al., 2015; Xun et al., 2020). Moreover, CXCR1 and CXCR2 expressed on immune cells can promote the recruitment of cancer microenvironment, thus affecting cancer invasion and metastasis (Xun et al., 2020). An experimental study showed that Tumor Necrosis Factor (TNF) augments CXCR2 to promote the progression of RCC leading to poor prognosis (Sun et al., 2016). However, our findings are not in compliance with findings from previous research, and the mechanism of CXCR1/2 involved in the regulation of ccRCC progression remains unclear. We speculate that CXCR1/2 may achieve an anti-tumor effect by increasing immune cell infiltration into the sites of tumor cells. In terms of prognosis, we found that high mRNA expression of CXCR2 and 4CpGs of CXCR2 were related to a favorable prognosis in ccRCC. Although CXCR1 was not significantly related to the prognosis of ccRCC, 7 CpGs of CXCR1 were correlated with significant OS time. Given the current studies on CXCR1 or CXCR2 in ccRCC remained sparse, CXCR1 and CXCR2 are worthy of further research.

We found that CXCR3 significantly up-regulated in ccRCC compared with normal samples, and patients with advanced stages or grades tended to express higher mRNA expression of CXCR3. Moreover, high mRNA expression CXCR3 was correlated with poor prognosis in ccRCC, and one CpG (cg17678039) of CXCR3 was related to favorable survival. Previous research has shown that overexpression of CXCR3 in cancer cells (e.g., breast cancer, gastric cancer cells) can promote cancer proliferation, migration, metastasis, and angiogenesis leading to poor clinical prognosis (Zhou et al., 2016; Bronger et al., 2017). Also, TNF-α could strongly up-regulate CXCR3 to facilitate invasion and metastasis of renal carcinoma, and high expression of CXCR3 was associated with poor prognosis (Sun et al., 2016). Besides, CXCR3 was found to be expressed by immune cells (macrophages, T cells, and dendritic cells), and CXCR3+leukocytes could be differentiated and recruited through paracrine signals to induce or inhibit cancer growth (Yang et al., 2011; Chen F. et al., 2019).

CXCR4 was highly expressed in ccRCC in both ONCOMINE database and TCGA dataset. CXCR4 is reported to be overexpressed in RCC and participate in the metastatic process (D'Alterio et al., 2010), and CXCR4 expression was related to advanced disease (Wehler et al., 2008). CXCR4 was previously described as a prognostic indicator of RCC, and high CXCR4 expression was associated with poor OS in ccRCC (Staller et al., 2003). In our study, an association was observed between high CXCR4 expression and worse prognosis for OS time, which was consistent with the previous research, and 15 CpGs of CXCR4 have also been found to be related to OS time. Recently, CXCR4 has been an emerging target for ccRCC treatment, and its antagonist could have a potential therapeutic effect against ccRCC (Song, 2017). High plasmacytoid dendritic cells could promote the expression of CXCR4 by TNF-α/NF-κB pathway (Gadalla et al., 2019), and blocking CXCR4 could effectively inhibit cancer progression and enhanced the curative effect of immune checkpoint blockers by up-regulating Treg cells infiltration (Chen I. X. et al., 2019).

CXCR5 was highly expressed in ccRCC in TCGA dataset, and patients with advanced stages or grades tended to express higher mRNA expression of CXCR5. Also, mRNA expression of CXCR5 and CXCR5 DNA methylation have been found to be correlated with OS time. Zheng et al. reported that expression of CXCR5 was higher in ccRCC cells compared with normal kidney cells, and ccRCC patients with high CXCR5 expression have a poor OS (Zheng et al., 2018), which was consistent with our results. Furthermore, it is reported that the CXCR5–CXCL13 axis could promote the progression of ccRCC (Zheng et al., 2018), and overexpression of CXCR5 facilitated tumor cell proliferation through JNK pathway in prostate cancer (El Haibi et al., 2010). On the contrary, suppressing CXCR5 could reduce cancer growth and liver metastasis (Meijer et al., 2006). CXCR5 can regulate B-cells, T cells, and dendritic cells in secondary lymphoid tissue (Schiffer et al., 2015), which could also contribute to tumor escape from the surveillance of immune system (Ding et al., 2015).

Our data showed that CXCR6 was highly expressed in ccRCC in both ONCOMINE and TCGA datasets, and the expression of CXCR6 was higher in ccRCC patients with late stages or advanced grades. Moreover, high expression of CXCR6 was associated with poor OS in ccRCC, but 3 CpGs of CXCR6 were related to favorable survival. Chang et al. reported that overexpression of CXCR6 was more likely to have a higher pathological grade and was a statistically significant predictor of worse OS in ccRCC patients (Chang et al., 2017). Overexpression of CXCR6 could facilitate cancer cell proliferation, invasion, and metastasis by regulating NF-κB pathway in prostate cancer (Kapur et al., 2019). Similar to other CXCRs, CXCR6–CXCL16 could recruit immune cells (e.g., CD4+ and CD8+ T cells) to cancer sites to affect cancer progression, but the cell-specific functions of CXCR6 remain largely unclear (Xun et al., 2020).

CXCR7, better known as ACKR3, was significantly overexpressed in ccRCC compared with normal kidney samples in ONCOMINE and TCGA datasets. Although OS tended to be shorter in CXCR7 low expression group, the difference was not statistically significant. In addition, CXCR7 DNA methylation have been found to be associated with the OS of ccRCC patients. Previous research showed that CRCX7 was associated with renal development and was up-regulated in about 50% of RCC (Mahmoodi et al., 2017). The expression of CXCR7 also significantly correlated with the lymph nodes status (D'Alterio et al., 2010). Our data indicated a correlation between CXCR4 and CXCR7, and a study has established that the combined evaluation of CXCR4 and CXCR7 is a valuable prognostic indicator for RCC patients (D'Alterio et al., 2010). Evidence demonstrated that overexpression of CXCR7 could regulate the secretion of VEGF to promote tumor growth and angiogenesis (Chen et al., 2016). At present, the biological functions of cancer-related CXCR7+ immune cells have not been intensively studied, but we found that the expression of CXCR7 was related to CD8+ T cell, CD4+ T cell, neutrophil and dendritic cell in ccRCC microenvironment.

To explore the potential mechanism of CXCR in ccRCC, functional enrichment analyses were performed, and the results revealed that CXCRs and their similar genes involved in the pathways related to tumor, angiogenesis, immune, and inflammatory responses. According to the results of PPI network, CXCRs mostly interact with CXCL1, CXCL6, CXCL8, CCR5, CCR3, CCL5, and APLN, and those hub genes in the PPI network could play important roles in the development and progression of ccRCC. According to our results and a previous study, the interactions among CXCRs, CXCL1, and CCL5 may mediate the regulatory T cells' participation in ccRCC progression (Wang et al., 2019). Recently, APLN receptor (APLNR) was considered to be an essential gene for tumor immunotherapy, which can modulate the function of CD8+T cells, but the immunological effects of the APLN/APLNR axis in ccRCC remain unknown (Tolkach et al., 2019). On the basis of previous research and our current results, we speculate that CXCRs may interact with APLN/APLNR axis to regulate the immune state of the tumor microenvironment in ccRCC. However, the precise mechanisms underlying these are not fully clear, and there are still few studies focusing on the relationship between these hub genes and ccRCC. Given the results of previous literature and our study, we can conclude that CXCRs are expressed by both tumor and immune cells to regulate cancer growth, invasion, and metastasis by various pathways in the ccRCC microenvironment, and the exact molecular mechanisms underlying these regulations in ccRCC are not fully understood. In short, our results add to the evidence for the complexity of CXCR1-7 and their related biological functions, which may provide a valuable reference for the development of CXCRs-mediated targeted therapy. However, there were some limitations in this study. Firstly, our research lacked experimental verification. Besides, the limited sample size of several sub-groups of survival analyses and the potential patient heterogeneity may bias the results.

To sum up, CXCR4/6/7 are significantly highly expressed in ccRCC, and overexpression of seven CXCRs members was found to be correlated with tumor stages and pathological grades in patients with ccRCC. Additionally, we found that mRNA expression of CXCR3/4/5/6 and CpGs methylation in all CXCRs members were significantly associated with OS in ccRCC patients. Besides, genetic alterations in CXCRs were remarkably related to shorter OS and PFS in ccRCC patients. These findings suggested that CXCRs could be prognostic biomarkers of ccRCC patients. However, further research is needed to verify these results and facilitate the clinical application of CXCR family members in ccRCC.

## Data Availability Statement

The datasets generated for this study can be found in online repositories. The names of the repository/repositories and accession number(s) can be found in the article/Supplementary Material.

## Author Contributions

ZW and LP designed the study. YZ, XC, WT, and LH analyzed the data. ZW and YZ wrote the draft of the manuscript. LH and LP edited the manuscript. All authors read and approved the final manuscript.

## Conflict of Interest

The authors declare that the research was conducted in the absence of any commercial or financial relationships that could be construed as a potential conflict of interest.
