# Supplementary material for: Characterization of the Prognostic Values of the CXCR1-7 in Clear Cell Renal Cell Carcinoma (ccRCC) Microenvironment
Source: Front Mol Biosci. 2020 Nov 25;7:601206. doi: 10.3389/fmolb.2020.601206 (PMC7724088; doi:10.3389/fmolb.2020.601206)
Supplement: Supplementary file 1 [file Table_1.DOCX]

Supplementary Material

# Supplementary Figures


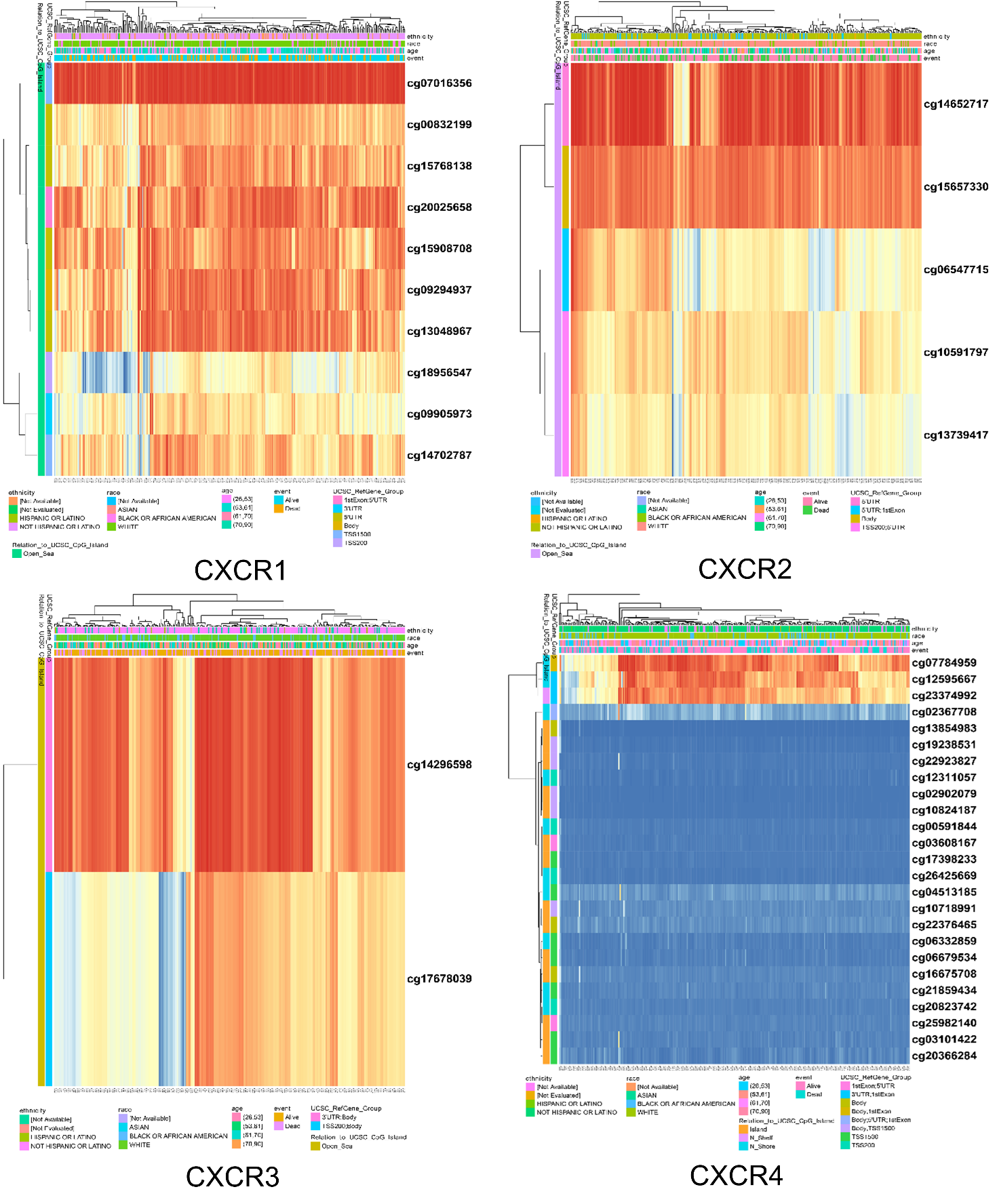


**Supplementary Figure 1.** DNA methylation of CXCR1-4 in MethSurv. The DNA methylation levels of CXCR1/2/3/4. Red to blue: high expression to low expression.


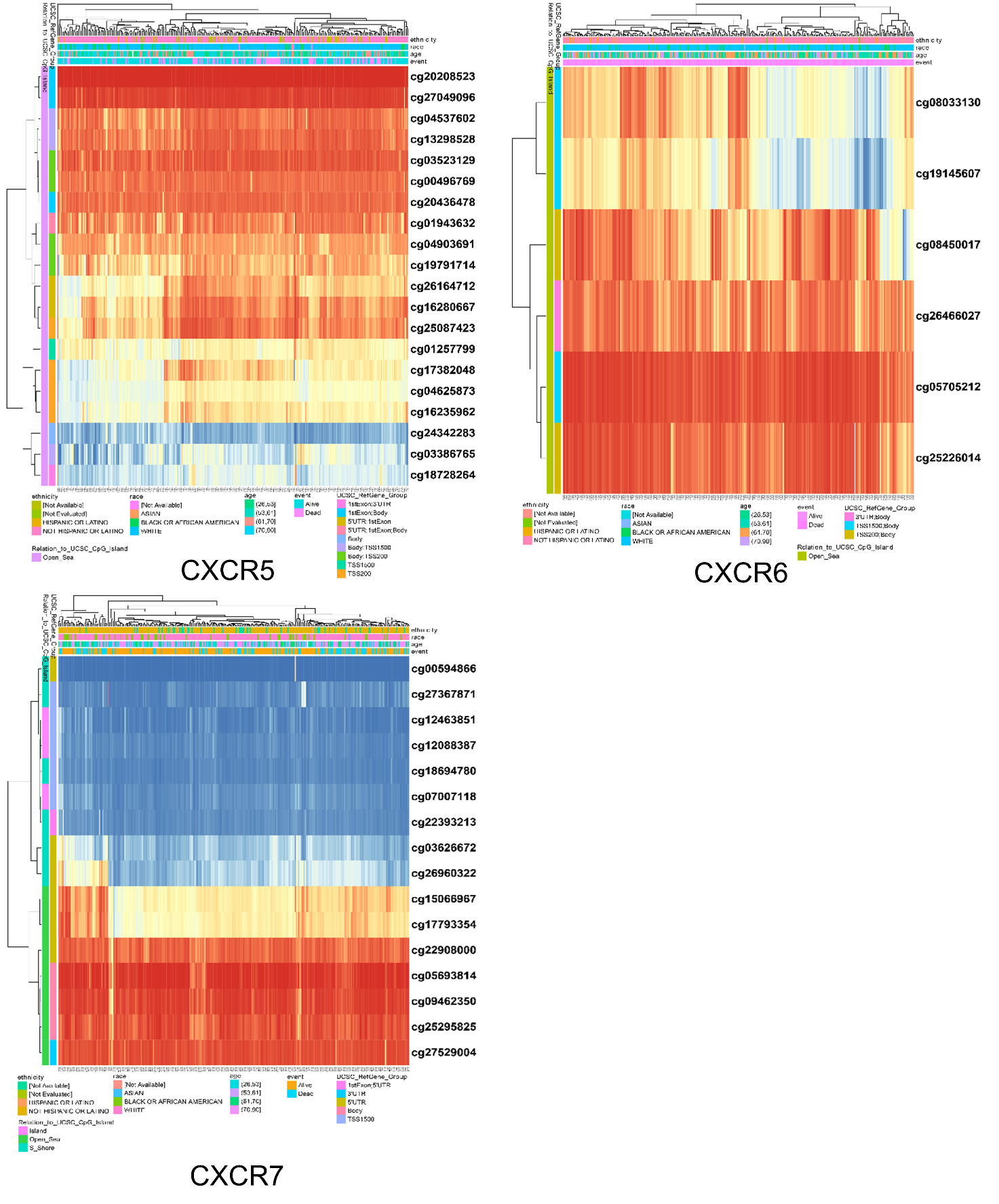
**Supplementary Figure 2.** DNA methylation of CXCR5-CXCR7 in MethSurv. The DNA methylation levels of CXCR5/6/7. Red to blue: high expression to low expression.

##
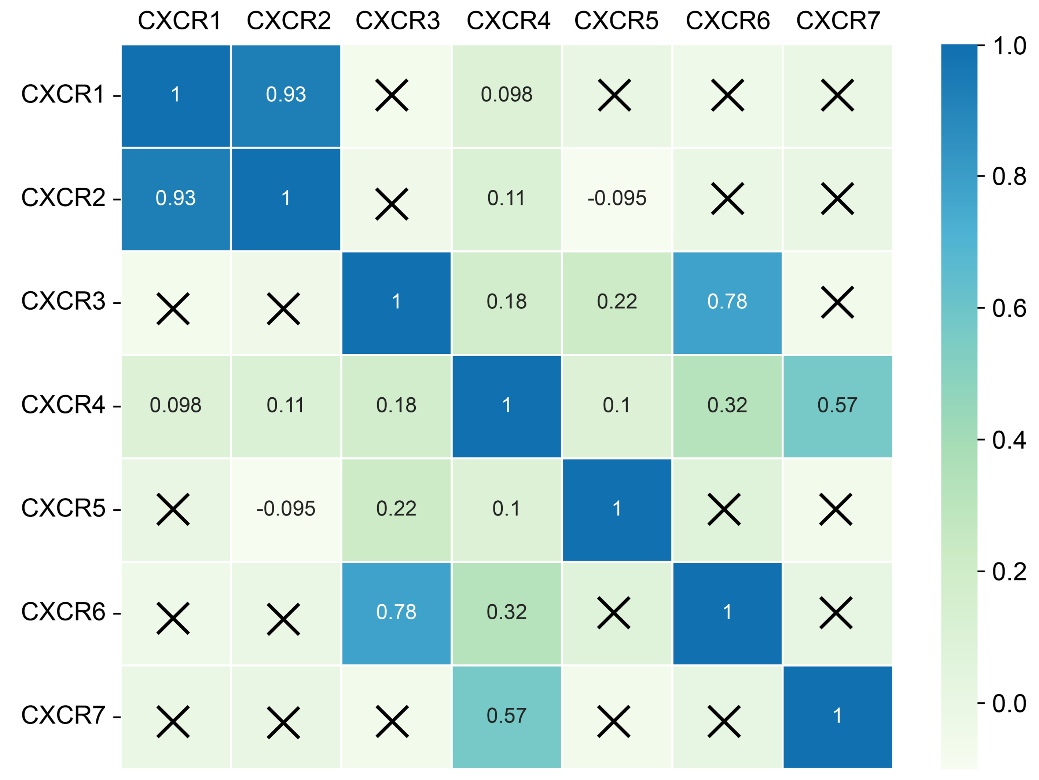


**Supplementary Figure 3.** Pearson correlation of CXCRs family members. The cross indicates p>0.05.

# Supplementary Tables

**Supplementary Table 1.** Top 20 similar genes of each CXCR family member (GEPIA).

| CXCRs | Similar genes extracted from GEPIA |
| --- | --- |
| CXCR1 | CXCR2, CMTM2, CEACAM3, CTC-510F12.4, MMP25, Y_RNA, IGHVII-1-1, AC074338.5, ADGRG3, CXCL1, FCGR3B, CSF3R, CXCL6, PADI4, KB-1980E6.3, RP11-126O22.1, RP11-126O22.5, S100P, RP11-126O22.11, FFAR2 |
| CXCR2 | CXCR1, FCGR3B, CEACAM3, CMTM2, ADGRE3, CSF3R, MMP25, CTC-510F12.4, ADGRG3, IGHVII-1-1, AC074338.5, Y_RNA, PADI4, CXCL6, CXCL1, CXCL8, RP11-126O22.1, KB-1980E6.3, CCR3, FFAR2 |
| CXCR3 | SIT1, TRAC, UBASH3A, CD3E, CD27, CD2, LTA, SIRPG, PVRIG, CD3D, LCK, CCL5, FASLG, SLA2, IL2RG, TRBC2, PTPRCAP, CORO1A, CD8A, JAKMIP1 |
| CXCR4 | CXCR7, ARHGAP25, PDE4B, FAM49A, RGS1, RIN3, CD69, IFI16, TNFRSF1B, C10orf10, GGTA1P, ANGPT2, GRAP, VIM, KCNJ2, LAMA4, PALD1, BTG1, SMAP2, EOGT |
| CXCR5 | CTD-2369P2.12, CITF22-1A6.3, RP4-761J14.8, AL513523.2, RP11-746M1.1, CTC-444N24.8, RP11-24N18.1, SART1, RP11-294J22.6, RP11-697E22.1, RP11-96D1.11, AC074212.5, RP11-196H14.3, ZACN, RP11-256P1.1, RP11-152H18.3, RP11-63M22.2, RP11-500C12.1, SIPA1, HCG25 |
| CXCR6 | CD2, SLA2, CCR5, CD96, SLAMF6, IL2RG, SNX20, CD3E, LCK, UBASH3A, TIGIT, SASH3, IKZF3, CD53, SH2D1A, TRAT1, IL12RB1, TRAC, RAC2, ICOS |
| CXCR7 | ANGPT2, DLL4, SEMA6B, MCAM, KCNJ2, TNFAIP8L1, INHBB, RHOJ, GRAP, UNC5B, LAMA4, CDH5, CXorf36, FAM43A, APCDD1, GJA1, ACE, NR5A2, SLC1A4, APLN |
